# Tyrosine kinase inhibitors in metastatic pheochromocytomas and paragangliomas: an updated systematic review and meta-analysis

**DOI:** 10.1530/ERC-26-0201

**Published:** 2026-10-27

**Authors:** Xiaowen Xu, Quanheng Liu, Shihui Xu, Awan Hasham Ikram, Herve Pasteur Ndikuriyo, Yizhou Wang, Kai Cheng, Zhi Li, Junjie Chen, Minghao Li, Qiao Xiao, Longfei Liu

**Affiliations:** ^1^ Department of Urology, Xiangya Hospital, Central South University, Changsha, China; ^2^ National Clinical Research Center for Geriatric Disorders (Xiangya Hospital), Central South University, Changsha, China; ^3^ Department of Urology, Jiangxi Provincial People’s Hospital, Nanchang, China; ^4^ Department of Physiology, College of Medicine and Health, University College Cork, Cork, Ireland

**Keywords:** pheochromocytomas and paragangliomas, tyrosine kinase inhibitors, metastasis, *SDHB*, meta-analysis

## Abstract

Therapeutic options for metastatic pheochromocytomas and paragangliomas (mPPGLs) remain limited. Recent studies indicate that tyrosine kinase inhibitors (TKIs) offer a promising treatment approach. This study aims to evaluate the efficacy and safety of TKIs for mPPGLs based on available evidence. A systematic search for studies investigating the treatment of mPPGLs with TKIs was conducted across PubMed, Embase, and Web of Science from database inception to February 2026. Data were synthesized using fixed- or random-effects meta-analysis model. Subgroup analysis was conducted based on the status of the *SDHB* gene and subtypes of TKIs. A total of 11 studies, comprising 4 observational and 7 interventional studies, with 246 patients involved. The stable disease rate, objective response rate (ORR), and disease control rate (DCR) were 51.8% (95% CI: 44.6–59.1%), 27.7% (95% CI: 21.6–34.3%), and 84.2% (95% CI: 74.8–91.9%), respectively. The proportion of discontinuation due to adverse events was 11.3% (95% CI: 5.4–18.7%). The combined median progression-free survival and overall survival were 13.4 (95% CI: 7.9–14.7) and 26.7 (95% CI: 24.9–49.4) months. Pooled ORR (36.8% (95% CI: 21.1–53.8%) vs 20.1% (95% CI: 10.8–31.0%), *P* = 0.06) and DCR (92.2% (95% CI: 79.6–99.7%) vs 78.5% (95% CI: 57.3–94.5%); *P* = 0.269) were higher in patients with *SDHB*-related mPPGL than in those with non-*SDHB* mPPGL, although the differences were not statistically significant. Notably, sunitinib showed no therapeutic superiority over other TKI subtypes, with lower pooled ORR (21.2% (95% CI: 14.0–29.4%) vs 36.9% (95% CI: 26.9–47.4%); *P* = 0.04) and DCR (72.6% (95% CI: 63.9–80.6%) vs 94.5% (95% CI: 87.2–99.3%); *P* = 0.001). Consequently, TKIs show promising efficacy and manageable adverse event rates in mPPGL, supporting TKIs as components of the systematic treatment.

## Introduction

Pheochromocytomas and paragangliomas (PPGLs) are rare neuroendocrine tumors arising from chromaffin cells in the adrenal medulla and from sympathetic or parasympathetic ganglia, respectively (1, 2). Metastatic disease is diagnosed in approximately 3–15% of cases (3, 4, 5, 6). Systemic radiotherapies, such as ^177^Lu-DOTATATE peptide receptor radionuclide therapy (PRRT) and ^131^I-meta-iodobenzylguanidine (MIBG), are available for tumors with somatostatin receptor (SSTR) positivity or MIBG avidity (7, 8, 9, 10). Chemotherapies using agents such as cyclophosphamide, vincristine, dacarbazine (CVD), and temozolomide are typically employed in patients with rapidly progressing tumors or those unresponsive to radiotherapies (11, 12, 13, 14). However, the efficacy of these treatments is suboptimal, and the prognosis for patients with metastatic PPGL (mPPGL) is poor, with a 5-year overall survival rate ranging from about 30 to 60% (6, 15).

When focusing on the development of metastatic disease, the stabilization of hypoxia-inducible factor 2 alpha (HIF-2α) and the activation of hypoxic signaling pathways are critical, particularly in tumors with pathogenic variants (PVs) in cluster 1 genotype, which is also termed the pseudo-hypoxic cluster (16, 17). The accumulation of HIF-2α drives angiogenesis by activating transcription of vascular endothelial growth factor (*VEGF*) and platelet-derived growth factor (*PDGF*), among other cellular processes (16, 18, 19, 20). Elevated levels of VEGF and VEGF receptor (VEGFR) have been observed in mPPGLs, especially in tumors harboring PVs in succinate dehydrogenase subunit B (*SDHB*), the gene most related to metastasis (21, 22, 23, 24, 25). This link between angiogenesis and metastatic progression provides potential targets for antitumor therapies (26).

Tyrosine kinase inhibitors (TKIs) are multi-targeted agents that exert antitumor effects by inhibiting receptors involved in angiogenesis, such as VEGFR, PDGFR, and fibroblast growth factor receptor (FGFR) (11, 27, 28). The potential efficacy of these drugs against mPPGLs is not only consistent with the biological features of tumors mentioned above but also with recent prospective studies and clinical trials that have shown TKIs to be a reliable systemic treatment option for inoperable or progressive diseases (29, 30, 31, 32, 33). There is high-level evidence from randomized controlled trials in the treatment of sunitinib for progressive mPPGLs (29, 31, 34, 35, 36). The National Comprehensive Cancer Network (NCCN) has also included sunitinib in the treatment guidelines for locally unresectable PPGLs and distant metastases, as per the version 1.2022 update (37). Besides, cabozantinib, axitinib, anlotinib, and other subtypes of TKIs have also shown encouraging efficiency in treating mPPGLs (30, 32, 38, 39, 40). However, tumor response rates vary across studies, and the exact effects of TKIs on mPPGLs remain unclear. Including TKIs, the optimal selection and flow of systemic therapies for PPGLs remain controversial across different genetic backgrounds and disease stages (41, 42, 43). Therefore, it is important to systematically evaluate these aspects from a clinical perspective.

In this study, we conducted a meta-analysis and systematic review to evaluate the efficacy and safety of various subtypes of TKI therapy for different genotypes of mPPGLs and to clarify their potential role in the current systemic therapy concept. Importantly, no randomized clinical trials have directly compared the available therapeutic options head-to-head. Therefore, differences in efficacy across treatments identified in this review should be interpreted cautiously and should not be regarded as evidence of comparative efficacy.

## Methods

This evidence-based analysis was conducted in accordance with the Preferred Reporting Items for Systematic Reviews and Meta-Analysis (PRISMA) statement (44) (Supplementary Materials 2 (see section on Supplementary materials given at the end of the article)) and the Assessing the Methodological Quality of Systematic Reviews 2 (AMSTAR2) guidelines (45) (Supplementary Materials 3). The study protocol was preregistered in PROSPERO (CRD42024579595).

### Search strategy and selection criteria

A systematic search was performed across the PubMed, Embase, and Web of Science databases up to February 28, 2026. The search included keywords such as ‘PPGLs’, ‘pheochromocytomas’, or ‘paragangliomas’, along with the names of six TKIs – sunitinib, cabozantinib, axitinib, pazopanib, lenvatinib, and anlotinib – based on a review of targeted therapies for PPGLs by Wang *et al.* (11), and related synonyms, following the ‘PICOS’ principles. The detailed search strategy is provided in Supplementary Table 1. To ensure a comprehensive search, studies and reviews were considered without limitations on publication type, language, region, or references. Given the rarity of mPPGL and the limited number of studies evaluating TKIs, we adopted broad eligibility criteria to capture the available clinical data. Studies were included if they met the following criteria: i) study design as randomized controlled trial (RCT), cohort study, case–control study, or single-arm study; ii) inclusion of patients with mPPGL, defined as the presence of distant metastases at sites devoid of chromaffin cells (e.g. bone or lymph nodes); iii) use of at least one of the six TKIs as the intervention; iv) availability of data on tumor response and survival, including complete response (CR), partial response (PR), stable disease (SD), progressive disease (PD), objective response rate (ORR), disease control rate (DCR), overall survival (OS), progression-free survival (PFS) at 12 months, or median OS and PFS, based on Response Evaluation Criteria in Solid Tumors (RECIST) version 1.1. Exclusion criteria included: i) studies without original data, such as editorials, comments, reviews, case reports, and protocols; ii) studies reporting fewer than five evaluable patients; iii) *in vitro* studies; iv) repeated studies describing the same cohort; v) studies with duplicate publications from the same research group.

### Data extraction and quality assessment

Data extraction was performed independently by two investigators (XX and QL). In case of disagreement, a third investigator (QX) was consulted to make the final determination.

The information extracted as baseline characteristics included the first author, year of publication, study design, total number of participants, doses of TKIs, demographic details of patients, treatment duration, and follow-up information. We also extracted and descriptively summarized the requirements of each study for disease progression before TKI treatment and the available baseline prognostic characteristics (Supplementary Table 2). However, the published reports did not provide sufficient longitudinal progression and prognostic data for individual patients to reconstruct their pretreatment disease course or adjust the analyses for baseline disease aggressiveness.

Endpoint data extracted encompassed tumor response (CR, PR, SD, PD, ORR, and DCR), survival data (OS and PFS at 12 months post-TKI therapy, median OS, and median PFS), and the incidence of adverse events (AEs). The AE data included the most commonly reported AEs, the number of patients with different severity grades, and the number of patients who discontinued or reduced their dosage due to AEs.

Tumor response evaluation was extracted as reported by the original included studies, which described these responses as assessed according to RECIST version 1.1 (46). For patients with bone lesions or non-measurable disease, some studies, including Ayala-Ramirez *et al.* and Jimenez *et al.*, used PET/CT based assessments of lesions. Because the available data were limited, we could not review the imaging data or reclassify individual patient responses. CR was defined as the complete disappearance of all target lesions, with any pathological lymph nodes (whether classified as target or non-target) reducing in the short axis measurement to less than 10 mm. PR was defined as a reduction of at least 30% in the sum of diameters of target lesions compared with baseline. PD was defined by a minimum increase of 20% in the sum of target lesions’ diameters relative to the smallest sum recorded during the study, with an absolute increase of at least 5 mm in addition to the 20% increase. SD was defined as the condition where the reduction did not meet PR criteria and the increase did not meet PD criteria, with the smallest recorded sum of diameters during the study serving as the reference. ORR was calculated as the percentage of patients achieving PR, while DCR was the sum of the percentages of patients achieving both PR and SD.

The quality of single-arm, interventional studies was assessed using the Methodological Index for Non-Randomized Studies (MINORS) scale (47), which includes eight criteria, each evaluated on a three-level scale: 0 (not reported), 1 (reported but inadequate), or 2 (reported and adequate).

The Joanna Briggs Institute (JBI) tools (48) were employed to assess the quality of case series observational studies. Each of the ten criteria was rated as ‘yes’, ‘no’, ‘unclear’, or ‘not applicable’.

Two investigators independently assessed the quality and level of evidence for eligible studies. Any discrepancies were resolved through discussion.

### Statistical analysis

Evidence synthesis was conducted using Stata 14.0 (StataCorp, USA) and R (version 4.1.1). Heterogeneity across studies was assessed using Cochran’s Q test and the inconsistency index (*I*^2^), applying the ‘metaprop’ function in the ‘meta’ package. A *P*-value <0.05 for Cochran’s Q test or *I*^2^ > 50% indicated significant heterogeneity. In cases of significant heterogeneity, a random-effects model was employed to calculate combined proportions, while a fixed-effects model was used otherwise. For median PFS and OS, the ‘pool.med’ function in the ‘metamedian’ package of R (version 4.1.1) was applied, utilizing the weighted median of medians method. Forest plots were generated using the ‘forestplot’ function.

Potential small-study effects were explored by visual inspection of funnel plots generated using the ‘metafunnel’ command and by testing funnel-plot asymmetry with Egger’s test using the ‘metabias’ command in Stata. Leave-one-out sensitivity analysis was performed using the ‘metaninf’ function to assess the influence of individual studies on the combined results for outcomes exhibiting significant heterogeneity. Subgroup analysis was conducted using the ‘metaprop’ function to compare differences in efficacy and safety across subgroups. All metrics were reported with 95% confidence intervals (CIs), and *P* < 0.05 was considered statistically significant.

## Results

### Study selection and characteristics

The PRISMA flowchart outlines the literature screening process in detail (Fig. 1). A total of 2,304 relevant articles were identified from PubMed (*n* = 320), Embase (*n* = 1,021), and Web of Science (*n* = 963) through the systematic search. After removing duplicates, 1,552 titles and abstracts were reviewed. The full texts of 42 potentially relevant records were further examined, and 11 studies (29, 30, 31, 32, 34, 35, 36, 38, 39, 40, 49) were ultimately included in the meta-analysis: 4 single-arm observational studies and 7 interventional studies (6 single-arm interventional studies and 1 RCT) (Table 1), which involved a total of 246 patients.

**Figure 1:**
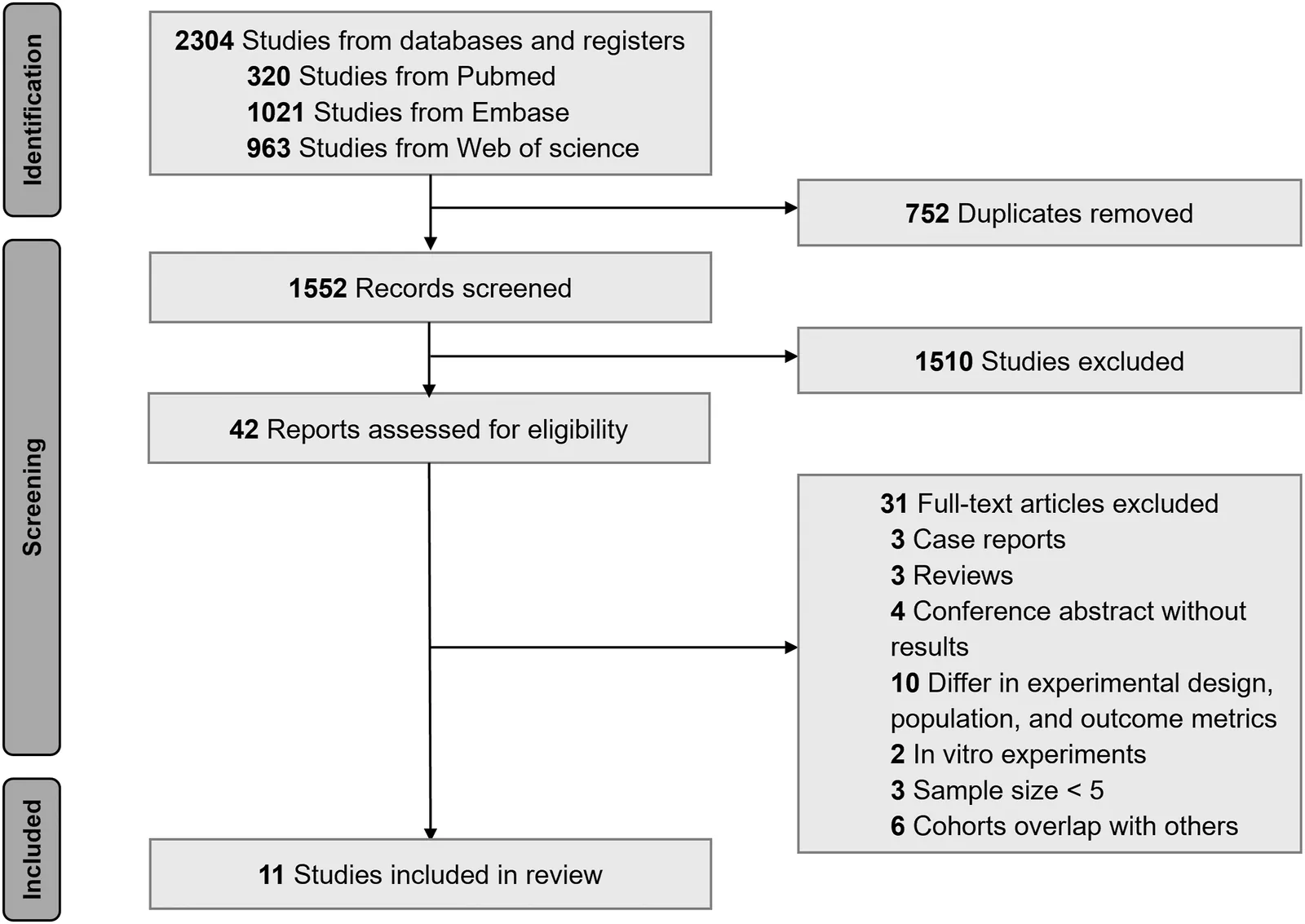
Flow diagram of study search and selection. After removing duplicates, screening abstracts, and evaluating full-text eligibility from 2,304 initial records, 11 studies were ultimately included.

**Table 1:** Baseline characteristics of included studies.

No.	First author	Year	Study type	NCT number	Number of patients	Age, years (range)	Primary tumor sites	Sex, male, No. (%)	Genetic background, No.	Type and dose of TKI	Median duration of treatment, months	Median duration of follow-up
1	Ayala-Ramirez (38)	2012	Observational	NA	17	46.5 (14–63) mean (range)	PGL: 6 PHEO: 11	9 (52.9)	*SDHB*: 8 *VHL*: 1	Sunitinib, most with 50 mg/d initially, 4 weeks (w) on and 2 weeks off; some decreased to 37.5 mg/d, 3 weeks on and 1 week off due to AEs; 3 with 37.5 mg/d initially	NA	36 months
2	Baudin (32)	2024	RCT	NCT01371201	39	58 (50–65) median (IQR)	NA	25 (64.1)	*SDHB*: 13	Sunitinib, 37.5 mg/d, could decrease to 25 mg or 12.5 mg/d if AE occurs	12 (IQR, 4–18)	29.7 months (IQR, 18.4–37.0)
3	Fojo (44)	2024	Interventional	NCT03839498	19	NA	NA	NA	NA	Axitinib, 5 mg twice daily initially on a 28-day cycle; patients were evaluated for dose adjustments to levels of 2, 3, 7, and 10 mg bid as options	9.5	NA
4	Jimenez (33)	2024	Interventional	NCT02302833	17	52 (42–56) median (IQR)	PGL: 7 PHEO: 10	13 (76.5)	*SDHB*: 5	Cabozantinib, 60 mg/d; dose was titrated down to 40 mg or 20 mg/d based on the severity of AEs and side effects	NA	25 months (IQR, 18–49)
5	Nasca (34)	2024	Interventional	EudraCT number: 2011–002632-99	50	56 (43–61) median (IQR)	NA	33 (66)	Pseudo-hypoxia cluster: 17 Kinase signaling cluster: 7 Negative: 7	Sunitinib, 50 mg/d daily for 4 weeks followed by a 2-week rest period until progressive disease, unacceptable toxicity, or informed consent withdrawal	14.0 (range, 1.4–94.5)	71.7 months (IQR, 35.4–100.1)
6	Nelson (39)	2022	Observational	NCT03008369	11	56 (44–69) mean (range)	PGL: 9 PHEO: 2	7 (63.6)	*SDHB*: 5	Lenvatinib, 20 mg/d initially, 28 days as a cycle; dose held/reduced due to AEs	14.7 (95% CI: 2.3-NA)	14.7 months
7	O’Kane (40)	2019	Interventional	NCT00843037	25	50 (17–79) median (range)	PGL: 11 PHEO: 14	14 (56)	*SDHB*: 5	Sunitinib, 50 mg/d orally, 4 weeks on and 2 weeks off as a cycle; doses decrease if intolerable AE occurs	12.4 (range, 1–88)	28 days after discontinuation
8	Park (41)	2024	Observational	NA	6	NA	NA	NA	NA	Sunitinib, 50 mg daily, 2 weeks on and 1 week off	NA	NA
9	Pichun (43)	2015	Interventional	NCT01967576	14	52.66 ± 11 mean ± SD	NA	10 (71.4)	NA	Axitinib, 5 mg twice daily initially on a 28-day cycle	NA	40.6 months
10	Tian (42)	2024	Observational	NA	19	NA	NA	NA	NA	Anlotinib, 10–12 mg/d on days 1–14, followed by a 7-day ceasing period (at least one course of treatment) until disease progression or intolerability	13.05 (range, 1.07–25.33)	13.58 months (range, 1.13–25.63)
11	Cui	2025	Interventional	NCT04860700	29	39.5 ± 12.6 mean ± SD	NA	18 (62.1)	*SDHB*: 13 *RET*: 3 *FH*: 1	Anlotinib hydrochloride 8–12 mg orally once daily on days 1–14	NA	NA

NA, not applicable; RCT, randomized controlled trial; TKI, tyrosine kinase inhibitor; PGL, paraganglioma; PHEO, pheochromocytoma; AEs, adverse events.

Two of the seven interventional studies received a score of one for prospective data collection, as the primary study design did not meet the expected outcomes. Of the 11 studies, 5 did not specify whether the endpoints were assessed on an intention-to-treat basis and were, thus, classified as ‘reported but inadequate’. Five studies reported a follow-up loss greater than 5%. One study did not include a prospective calculation of study size (Supplementary Table 3).

Of the 11 studies, 10 were available in full text, and 1 was a conference abstract. All studies were published between November 2012 and December 2025 and were written in English. Four studies were observational, and seven were interventional. The interventional arm of one RCT was analyzed separately, while the remaining ten studies were single-arm. Five studies evaluated the efficacy and safety of sunitinib, two for axitinib, two for anlotinib, and one each focused on lenvatinib and cabozantinib. In addition, seven studies reported germline PVs in susceptibility genes, with five providing detailed tumor response data.

### The efficacy of TKIs in mPPGLs

A total of 218 patients with mPPGL were included in the analysis. The proportion of patients achieving PR and SD following intervention ranged from 13 to 62.5% and 35.7–69.6%, respectively. The ORR was equivalent to the PR rate, as no patients achieved CR. The DCR ranged from 57.1 to 100% (Supplementary Table 4). The pooled percentages for PR, SD, ORR, and DCR were 27.7% (95% CI: 21.6–34.3%), 51.8% (95% CI: 44.6–59.1%), 27.7% (95% CI: 21.6–34.3%), and 84.2% (95% CI: 74.8–91.9%), respectively (Fig. 2A, B, C).

**Figure 2:**
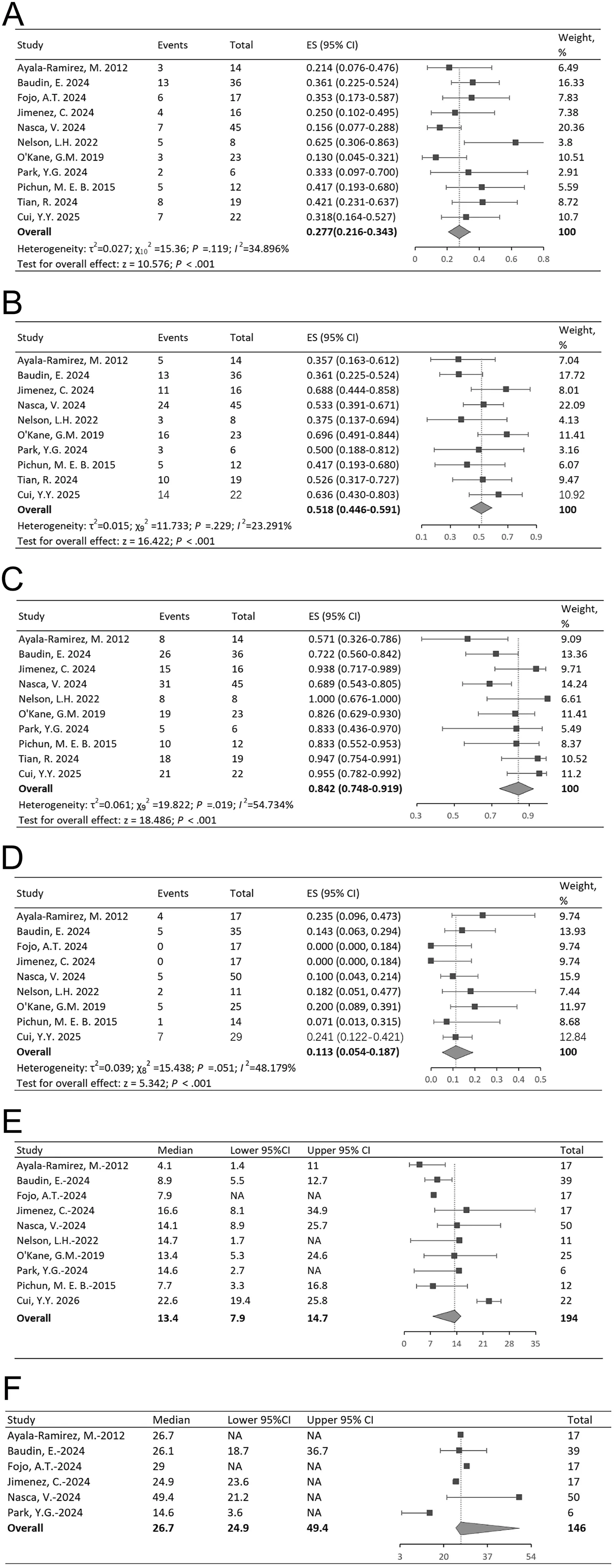
Efficacy and safety of TKIs for metastatic pheochromocytomas and paragangliomas. Forest plots display results of PR (A), SD (B), DCR (C), AEs (D), PFS (E), and OS (F) from individual studies and pooled percentages. The pooled estimates summarize outcomes across separate, predominantly single-arm studies and should not be interpreted as head-to-head comparisons or as evidence of comparative efficacy among individual TKIs or between TKIs and other therapeutic options. Squares and horizontal lines indicate the mean values and 95% confidence intervals of effect sizes, while diamonds illustrate the pooled effect estimates, with the width indicating the 95% confidence intervals. A dashed line represents the summary measure. NA, not applicable.

Ten studies reported median PFS, ranging from 4.1 to 22.6 months. The pooled median PFS was 13.4 (95% CI: 7.9–14.7) months. Median OS was reported in six studies involving 146 patients, with a range of 14.6–49.4 months. The pooled median OS was 26.7 (95% CI: 24.9–49.4) months (Fig. 2E and F). In addition, three studies reported 12-month OS ranging from 75.4 to 80.8%, while four studies showed 12-month PFS rates between 35.9 and 61.4% (Supplementary Table 4).

Two interventional studies assessed hormone response *via* urinary or plasma catecholamines and metanephrines. Among 47 patients, 35 were evaluable, and of these, 20 (57.14%) showed a hormonal response, defined as a reduction of at least 20% in one of the catecholamine markers over a 12-week period.

Two studies assessed the efficacy of TKIs in controlling abnormally elevated blood pressure. A total of 26 patients experienced hypertension exacerbation at the start of treatment. Of these, 61.5% (16/26) achieved blood pressure improvement through dose adjustments, defined as either reduced blood pressure or a reduction/discontinuation of antihypertensive drugs during treatment.

### Safety of TKI treatment

Nine studies, involving 215 patients, reported on the number of patients who discontinued or reduced their dosage due to AEs (Supplementary Table 5). The pooled proportion of discontinuation was 11.3% (95% CI: 5.4–18.7%) (Fig. 2D).

Common grade 1–2 AEs included hypertension, fatigue, vomiting, and palmar-plantar erythrodysesthesia syndrome (PPES). Grade 3–4 AEs primarily involved hypertension, fatigue or asthenia, and PPES. Two studies also reported serious grade 5 AEs in 3/39 and 1/50 patients, including amyotrophic lateral sclerosis (1), rectal bleeding (1), shortness of breath (1), and brain hemorrhage (1).

Thus, the most frequently reported AEs associated with TKI treatment were hypertension, fatigue, and PPES.

### TKI treatment for patients with mPPGL due to PVs in SDHB gene

Given the strong association between PVs in *SDHB* and metastatic disease, we compared the efficacy of TKIs in treating mPPGLs with and without PVs in *SDHB*. Five studies, involving 40 patients with *SDHB*-related tumors and 71 with non-*SDHB* tumors, were included (Supplementary Fig. 1). The pooled ORR for patients with *SDHB*-related tumors was 36.8% (95% CI: 21.1–53.8%) and suggested a numerically higher proportion compared with patients with non-*SDHB* PPGLs (20.1% (95% CI: 10.8–31.0%), *P* = 0.06), although without statistical difference. A similar trend was observed in the pooled DCR (92.2% (95% CI: 79.6–99.7%) vs 78.5% (95% CI: 57.3–94.5%); *P* = 0.269) (Fig. 3A and B). The pooled proportions of patients achieving SD were similar in both groups (52.6% (95% CI: 35.6–69.3%) vs 54.1% (95% CI: 31.0–76.5%); *P* = 0.907) (Fig. 3C).

**Figure 3:**
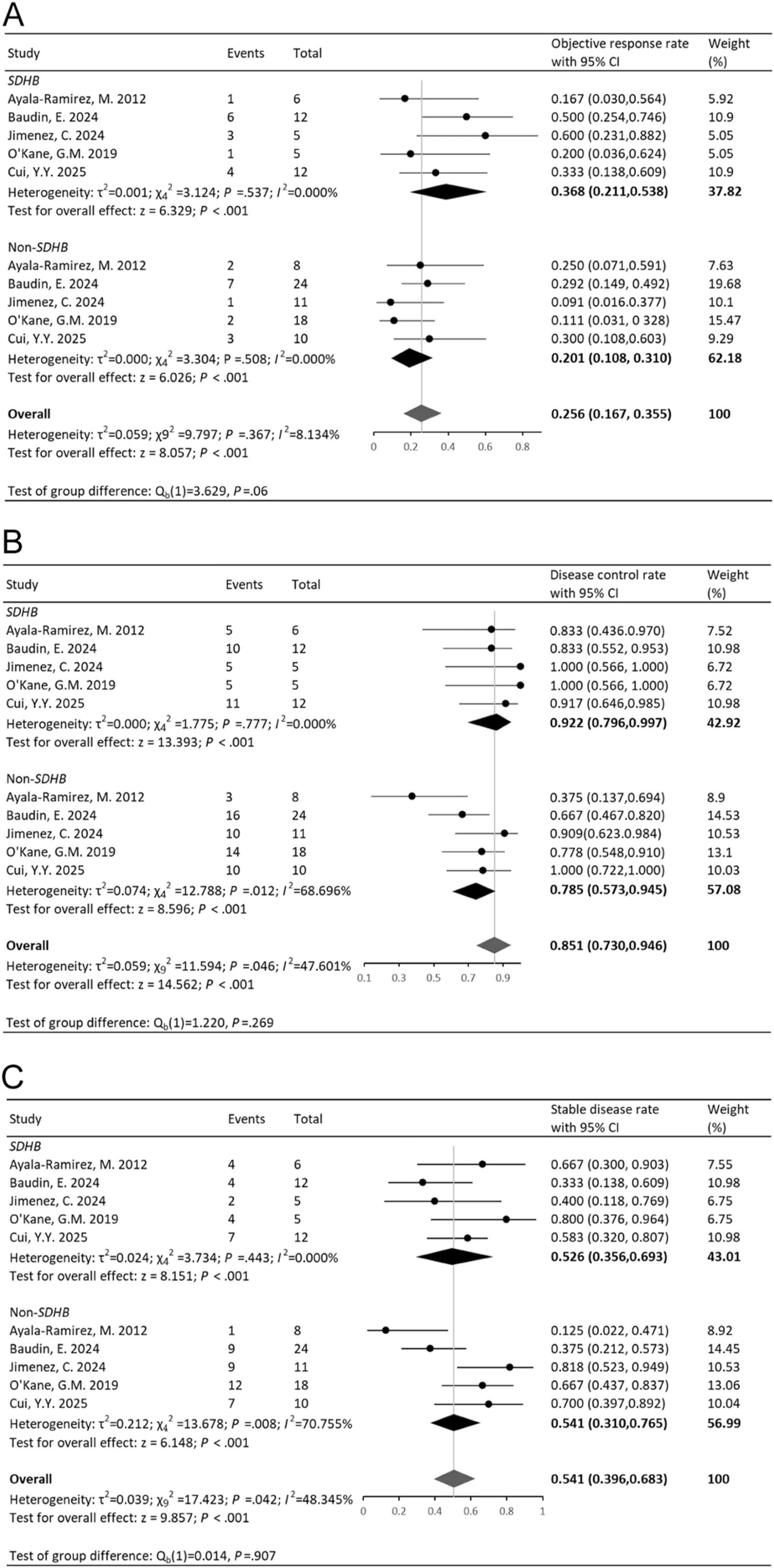
Subgroup analysis of tumor response based on state of PVs in *SDHB*. Forest plots display results of ORR (A), DCR (B), and SD (C) from individual studies and pooled percentages. Dots and horizontal lines indicate the mean values and 95% confidence intervals of effect sizes, while diamonds illustrate the pooled effect estimates, with the width indicating the 95% confidence intervals. A dashed line represents the summary measure. ORR, objective response rate; DCR, disease control rate; SD, stable disease.

### Comparison of the efficacy of different types of TKIs

Sunitinib was used in 5 of the 11 studies, with the remaining 6 utilizing other TKIs. Compared with other TKIs, sunitinib was associated with lower pooled ORR (21.2% (95% CI: 14.0–29.4%) vs 36.9% (95% CI: 26.9–47.4%); *P* = 0.04) and DCR (72.6% (95% CI: 63.9–80.6%) vs 94.5% (95% CI: 87.2–99.3%); *P* = 0.001) (Fig. 4A and B). Moreover, the incidence of treatment discontinuations due to AEs was twice in the sunitinib group compared with the other TKIs (14.4% (95% CI: 8.5–21.4%) vs 7.1% (95% CI: 0.0–20.6%); *P* = 0.351) (Fig. 4C).

**Figure 4:**
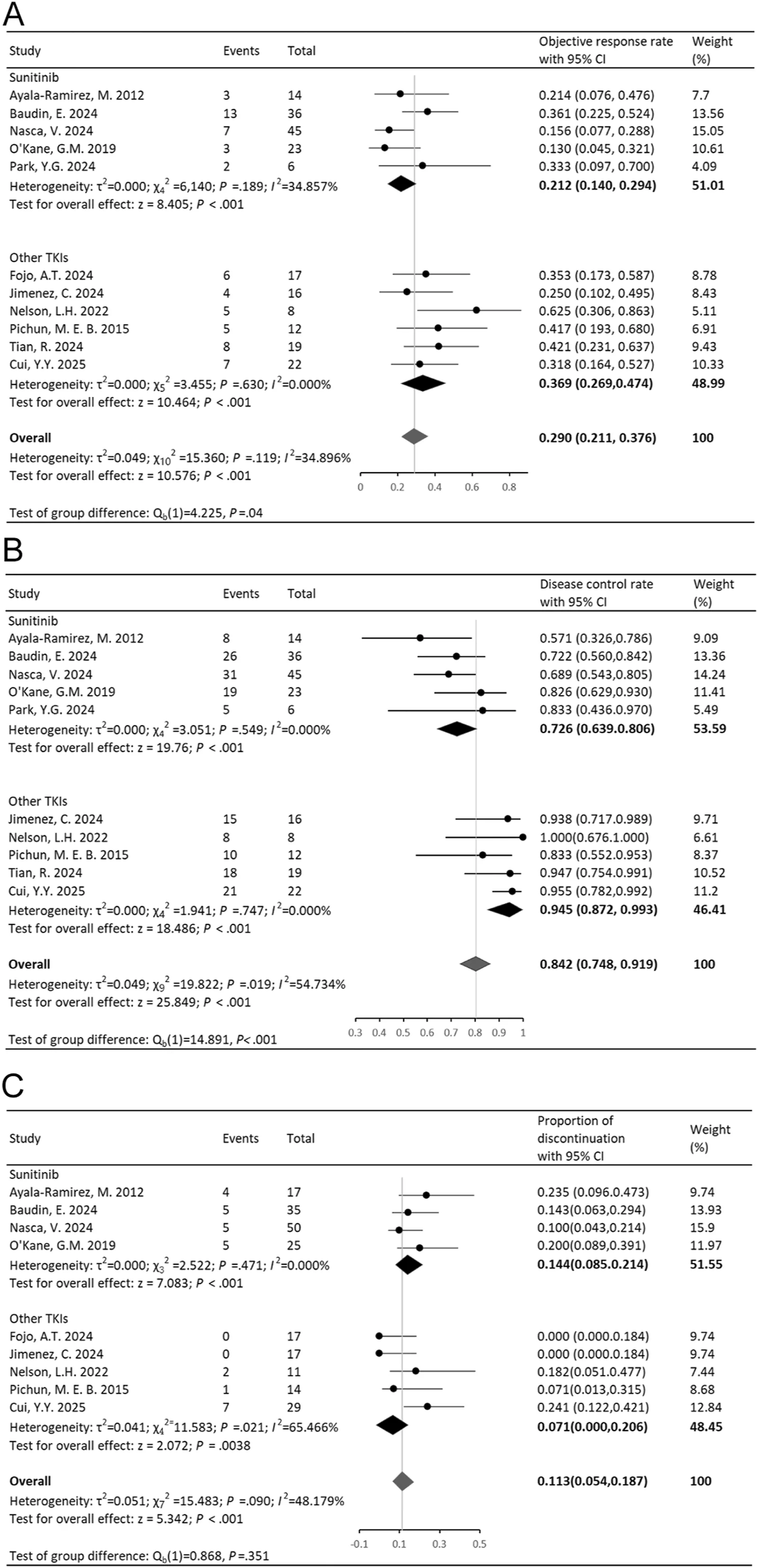
Subgroup analysis of tumor response and adverse events based on types of TKIs. Forest plots display results of ORR (A), DCR (B), and AEs (C) from individual studies, pooled results, and overall percentages. These subgroup estimates are based on indirect comparisons across separate studies and do not constitute randomized head-to-head evidence of comparative efficacy between sunitinib and other TKIs. Dots and horizontal lines indicate the mean values and 95% confidence intervals of effect sizes, while diamonds illustrate the pooled effect estimates, with the width indicating the 95% confidence intervals. The dashed line represents the summary measure. ORR, objective response rate; DCR, disease control rate; AEs, adverse events.

### Small-study effects, funnel-plot asymmetry bias and sensitivity analysis

Funnel plots were examined, and Egger’s tests did not detect statistically significant funnel-plot asymmetry for PR/ORR, SD, DCR, or AEs (*P* = 0.105, 0.864, 0.195, and 0.799, respectively) (Supplementary Figs 2, 3, 4, 5). Given the limited number and methodological heterogeneity of the included studies, the symmetry of the funnel plots does not provide sufficient evidence to conclude that we can totally rule out publication bias. Sensitivity analysis showed no significant changes in the results after excluding individual studies, indicating that none of the studies disproportionately impacted the overall findings (Supplementary Fig. 6).

## Discussion

The prognosis of patients with mPPGL is highly heterogeneous. In clinical practice, the importance of TKIs is not only based on the collected experience but also on the theoretical basis of their mechanism of action. Metastatic PPGL, especially pseudohypoxic tumors, have active angiogenesis, activating VEGF/VEGFR and PDGFR-related signaling pathways downstream of the HIF signaling pathway. This forms the theoretical basis for antiangiogenic therapy. This updated meta-analysis of 11 studies involving 246 patients indicates that, among 218 evaluable patients, the pooled ORR was 27.7%, the pooled SD accounted for 51.8%, the pooled DCR was 84.2%, the median PFS was 13.4 months, and the incidence of discontinuation due to toxicity was 11.3% in the analysis. Despite the limited number of evaluable patients, hormonal responses were observed in 57.14%, while blood pressure improved in 61.5%. This suggests TKIs can provide clinically meaningful disease stabilization and represent a promising therapeutic selection for patients with mPPGL.

However, we should consider with great caution whether TKIs can be established as a standard first-line therapy for all patients with mPPGL. In particular, the available evidence did not directly compare TKIs with radiopharmaceutical therapy or cytotoxic chemotherapy. Therefore, the available evidence cannot establish the relative efficacy or optimal sequencing of these treatment modalities. The studies we included and our analysis did not distinguish different prior treatments received before TKIs. As repeatedly emphasized in current studies and reviews, treatment should be individualized based on genetic background, tumor burden, disease progression, symptoms due to catecholamine excess or tumor compression, phenotype in functional imaging, comorbidities, general clinical condition, treatment tolerability and desires of patients, and so on (41, 50, 51, 52). Fischer *et al.* proposed an overall systemic therapy flow for mPPGLs based on the currently available clinical evidence and their applicability (42). For asymptomatic patients with low tumor volume, low tumor burden, and slow progression, active surveillance remains appropriate. Moreover, given the neuroendocrine nature of PPGLs, radiopharmaceutical therapy is often prioritized in tumors with high SSTR expression or strong MIBG avidity. It allows greater target specificity, sustained disease control, and acceptable toxicity (53, 54). By contrast, for lesions that are rapidly progressive, severely symptomatic, with large volume, or invading critical organs or tissues, cytotoxic chemotherapy (especially CVD or temozolomide-based regimens) may be preferred when rapid tumor reduction is required (50, 51). In this context, we support that TKIs should be considered as an important option for progressive, unresectable, or metastatic disease, especially when previous systemic therapies have been ineffective, imaging avidity is insufficient, or PRRT is not feasible. However, their use must be balanced against both previously experienced and subsequently potential treatment-related toxicities.

Adverse effects are a central aspect in the choice of therapy. Across included studies, the most frequently reported adverse reactions associated with TKIs are hypertension, fatigue, and PPES. Importantly, several toxicities particularly relevant in mPPGLs require special attention: worsening hypertension and catecholamine-related cardiovascular instability after the initiation of TKI treatment, proteinuria and hemorrhagic/ischemic events in patients with vascular complications, and other cumulative tolerability problems requiring dose reduction or discontinuation. For example, studies with sunitinib have shown that a significant proportion of patients discontinued treatment due to severe cardiovascular toxicity, stroke, myocardial infarction, cardiomyopathy, hemorrhage, or proteinuria (29, 31, 34, 35). In the recent anlotinib study, arterial hypertension occurred in 82.8% (24/29) of patients, and 24.1% (7/29) discontinued treatment at the early stage because of severe hormone-related AEs (32). These results highlight the importance of optimizing hemodynamic and endocrine management before TKI therapy, such as early and close monitoring of blood pressure and catecholamine excess and related symptoms from the initial phase of treatment, as well as the need for adequate α/β-adrenergic blockade when indicated.

Compared with the previous meta-analysis by Zhou *et al.* (33), our study included more recent prospective research evidence and provides a more up-to-date and deeper assessment of the efficacy of TKIs.

In the NCCN 2023 guidelines, sunitinib has been recommended for the treatment of locally unresectable or mPPGLs (37). Currently, sunitinib has a strong level of clinical evidence as the randomized, controlled trial FIRSTMAPPP demonstrated superior 12-month PFS, ORR, and DCR over placebo in progressive mPPGLs. Cabozantinib, axitinib, and anlotinib are also promising, with encouraging response rates and good disease control reported in prospective cohort studies. Interestingly, our subgroup analysis indicated that, compared with the pooled effects of other TKI subtypes, sunitinib was not superior and even appeared to be significantly inferior, with lower ORR (21.2 vs 36.9%, *P* = 0.04) and DCR (72.6 vs 94.5%, *P* = 0.001), and an approximately twofold higher rate of AEs (14.4 vs 7.1%, *P* = 0.351). However, these findings were derived from indirect, cross-study subgroup comparisons rather than randomized head-to-head trials of individual TKIs. They should, therefore, not be interpreted as demonstrating the absolute priority for any specific TKI in clinical practice. Potential bias may have arisen from differences across studies in patient characteristics, baseline and progression criteria, prior treatments, and treatment and monitoring strategies (Supplementary Table 2). For example, as a prospective RCT, FIRSTMAPPP may have enrolled patients with greater disease burden and applied closer monitoring and follow-up than observational studies, potentially producing more precise but also more conservative efficacy estimates. Nevertheless, the cross-study subgroup comparison was statistically feasible, within-subgroup heterogeneity was acceptable for the efficacy outcomes, and the between-subgroup differences reached statistical significance (Fig. 4). One possible explanation is that sunitinib primarily inhibits the VEGFR and PDGFR signaling pathways, targeting VEGFR1–3 and PDGFR-α/β, respectively (55). By contrast, lenvatinib and anlotinib can target VEGFR, PDGFR, and FGFR pathways (56, 57, 58), potentially leading to broader antiangiogenic effects and better disease control. Given the limited number of included studies, especially RCTs, differences among TKI subtypes should be interpreted cautiously. Accordingly, until more convincing data are available, sunitinib currently represents the TKI with the highest level of clinical evidence in practice. It is reasonable to regard other newer subtypes of TKIs as promising options for mPPGLs rather than definitively superior alternatives.

By studying genotypes, the use of TKIs may be more accurately determined. In our analysis, the pooled ORR and DCR for patients with *SDHB*-related tumors were higher than those for patients with non-*SDHB* tumors (36.8 vs 20.1%, 92.2 vs 78.5%, respectively). Although this pattern is compatible with the angiogenic biology of pseudohypoxic SDHB-deficient tumors, the differences between the two groups were not statistically significant (*P* = 0.06 and 0.269, respectively); this may be due to the relatively small sample size in the *SDHB* group (*n* = 40). In addition, the presence of non-*SDHB* mPPGLs may be attributed to PVs in other biologically heterogeneous genes, which could further reduce the statistical power. On the other hand, tumors driven by PVs in cluster 2 genes generally have a relatively lower tendency to metastasize. Although the use of TKIs is reasonable in view of the ability to target tyrosine kinases-associated signaling, treatment should still be individualized according to the aforementioned framework, with comprehensive consideration of the biochemical, imaging, and clinical phenotypes of patients (59). Since the evidence for metastatic diseases driven by PVs in cluster 3 genes remains very limited, the use of TKIs in this subgroup also requires individual assessment (60).

Accordingly, the potential efficacy and tolerability must be comprehensively considered before using TKIs. Uncontrollable catecholamine-related symptoms and hemodynamic manifestations, severe cardiovascular complications, active bleeding risk, and significant proteinuria are unlikely to be eligible for immediate TKI therapy. In these cases, other treatment options should be preferred if possible. Conversely, patients who require oral outpatient treatment, whose disease has advanced after PRRT, MIBG, or chemotherapy or for whom these treatments are unsuitable, especially patients with tumors harboring PVs in cluster 1 genes, could be particularly suitable candidates. Furthermore, the strategy of continuous sunitinib infusion at a low dose observed in the FIRSTMAPPP trial showed better efficacy and tolerability compared with the conventional intermittent dosing regimen of 50 mg (29). This suggests that careful dose selection, antihypertensive conditioning, and early dose adjustments form the basis for successful TKI therapy.

In addition, selective RET inhibitors represent a distinct subclass of TKIs and a genotype-directed strategy for *RET*-altered tumors. Unlike the antiangiogenic, multitarget TKIs evaluated in this meta-analysis, selective RET inhibition targets a defined molecular alteration. Alongside the HIF-2α inhibitor belzutifan, it highlights the potential of precision therapy guided by the underlying molecular drivers of PPGL. Although clinical evidence remains limited, the reported antitumor activity of selpercatinib and pralsetinib is noteworthy (61, 62, 63, 64). In the LIBRETTO-001 basket trial, four of six patients with *RET*-mutant pheochromocytoma achieved an objective response with selpercatinib, while the remaining two had stable disease (61). A case report of pralsetinib in MEN2B-associated pheochromocytoma described tumor shrinkage, biochemical improvement, and symptom relief (63). More recently, a pooled analysis of nine patients with advanced *RET*-altered pheochromocytoma, incorporating previously published cases and two additional patients, reported five PR, two CR, and two cases of SD (64). Thus, seven of nine patients achieved OR, with no patient classified as PD. These results suggest substantial, and in some cases durable, antitumor activity in this molecularly selected population. Nevertheless, these evidence derived from small, selected, and uncontrolled cohorts and did not permit a reliable comparison or pooling with multitarget TKIs of included studies in our meta-analysis. Dedicated prospective studies are needed to establish their efficacy and safety in *RET*-altered PPGL.

This study has several limitations. With the exception of one interventional RCT, all other included studies were single-arm and uncontrolled, and the number of patients available for subgroup analysis was limited. Egger’s tests did not detect statistically significant funnel-plot asymmetry, but publication bias could not be excluded. Although leave-one-out sensitivity analyses did not materially change the pooled estimates, indicating that no single study drove the overall findings, these procedures cannot eliminate selection bias, confounding, or differences in outcome ascertainment and follow-up across designs of included studies. Moreover, the enrolled patient populations may differ in baseline clinical status, metastatic burden and sites, prior treatments and lines of therapy, tumor secretory status, and molecular background. The included studies also differed in their requirements for disease progression before TKI initiation and in how pretreatment progression was documented. Thus, observed SD and longer PFS may partly reflect an intrinsically indolent disease course rather than TKI activity. Because detailed longitudinal progression data were unavailable for individual patients, we could not account for baseline disease aggressiveness when assessing TKI efficacy. This is a major limitation when interpreting and comparing therapeutic efficacy across non-randomized studies. Another potential concern is that, although the clinical trial registered by Fojo *et al.* required measurable disease at enrollment according to RECIST 1.1, it did not explicitly specify the criteria used to assess tumor response. This eligibility requirement suggests that RECIST 1.1 may also have been used for response assessment, but we cannot confirm this. Potential differences in tumor response, therefore, remain a possible source of heterogeneity.

These limitations may partly explain the variability in several outcomes across studies, such as ORRs ranging from 13.0 to 62.5% (*I*^2^ = 34.9%), DCRs from 57.1 to 100% (*I*^2^ = 54.7%), and median PFS from 4.1 to 22.6 months. Therefore, our conclusions on genotype-specific benefits and differences between TKI subtypes should be considered hypothetical rather than definitive. There is a compelling need for well-designed, large-scale clinical trials to further investigate, with the goal of optimizing treatment strategies for mPPGLs. Moreover, classification analyses should be conducted to account for confounding factors that may influence tumor response to TKI treatment, such as age, gender, and metastatic tumor activity, whether the tumor is sympathetic or parasympathetic PGL, and the baseline status and systemic treatments received by the included patients. Unfortunately, our study was unable to extract relevant data for further analysis.

In summary, TKIs were associated with objective responses in approximately one-quarter of evaluable patients and disease control in most patients, with a pooled median PFS of approximately one year. Therefore, in the context of genotype-guided personalized therapy, TKIs should be considered an important systemic treatment option for patients with mPPGLs. Future research should focus on biomarker-driven therapy prediction, stratified strategies by genotype and imaging manifestations, direct comparisons with radiopharmaceutical and chemotherapy strategies, and the development of rational combination therapies. These efforts are expected to provide clear guidelines regarding which patients should be treated early with TKIs, whether TKIs should be used first-line or only after other systemic therapies, and how toxicity can be minimized without compromising disease control.

## Supplementary materials







## Declaration of interest

The authors declare that there is no conflict of interest that could be perceived as prejudicing the impartiality of this work.

## Funding

This work was funded by the National Natural Science Foundation of Chinahttps://doi.org/10.13039/501100001809 (No. 82570929 and No. 82170806 to LL), the Natural Science Foundation of Hunan Provincehttps://doi.org/10.13039/501100004735 (No. 2023JJ30915 and No. 2025JJ20089 to LL), and the National Natural Science Foundation of China Young Scientist Fund (No. 82403632 to ML).

## Role of the funding source

The funder of the study had no role in study design, data collection, data analyses, data interpretation, or writing of the report.

## Author contribution statement

XX was involved in conceptualization, methodology, validation, investigation, data curation, and visualization, and wrote the original draft, as well as reviewed and edited the manuscript. QL dealt with conceptualization, methodology, software, investigation, data curation, and visualization, and wrote the original draft. SX and YW validated and visualized the study. AHI validated this study and wrote, reviewed, and edited this work. HPN wrote, reviewed, and edited this study. KC and ZL led the formal analysis and investigation. ML: supervised the study and acquired the funding. QX supervised and administered the project and wrote, reviewed, and edited this work. LL supervised and administered the project, acquired the funding, and wrote, reviewed, and edited this work.

## Data sharing statement

All authors had full access to all of the data in the study and take responsibility for the integrity of the data and the accuracy of the data analysis. The data and protocol are available from the corresponding author upon reasonable request.

## Ethical approval and informed consent

Not applicable.
