# Supplementary material for: Tyrosine kinase inhibitors in metastatic pheochromocytomas and paragangliomas: an updated systematic review and meta-analysis
Source: Endocr Relat Cancer. 2026 Oct 27;33(10):e260201. doi: 10.1530/ERC-26-0201 (PMC13726365; doi:10.1530/ERC-26-0201)
Supplement: Supplementary file 1 [file ERC-26-0201_supplementary_materials_1.pdf]

Tyrosine Kinase Inhibitors in Metastatic Pheochromocytomas and Paragangliomas: A Systematic Review and Meta-Analysis

Supplemental Materials

Supplementary Table 1: Search Strategy for Meta-analysis

Supplementary Table 2. Eligibility and baseline prognostic characteristics, prior treatment, line of therapy, and outcome analysis in the included studies

Supplementary Table 3. Quality Assessment

Supplementary Table 4. Tumor Response and Survival Data

Supplementary Table 5. Safety of TKI Treatment

Supplementary Figure 1. Forest Plot of Tumor Response for Patients with *SDHB* and with Non-*SDHB* PPGLs

Supplementary Figure 2. Funnel plot and Egger regression plot of PR/ORR

Supplementary Figure 3. Funnel plot and Egger regression plot of SD

Supplementary Figure 4. Funnel plot and Egger regression plot of DCR

Supplementary Figure 5. Funnel plot and Egger regression plot of AEs

Supplementary Figure 6. Sensitivity Analysis

Supplementary Table 1. Search Strategy for Meta-analysis

A. Search Terms and Queries

Topic		Number	Query
Patient/disease	Pheochromocytoma	#1	("Pheochromocytoma"[Mesh]) OR ((((((((Pheochromocytoma) OR (Pheochromocytomas)) OR (Pheochromocytoma, Extra-Adrenal)) OR (Pheochromocytomas)) OR (Extra-Adrenal Pheochromocytoma)) OR (Extra-Adrenal Pheochromocytomas)) OR (Pheochromocytoma, Extra Adrenal)) OR (Pheochromocytomas, Extra-Adrenal))
	Paraganglioma	#2	("Paraganglioma"[Mesh]) OR (((((((Paraganglioma) OR (Paragangliomas)) OR (Paragangliomata)) OR (Paragangliomatas)) OR (Paraganglioma, Gangliocytic)) OR (Gangliocytic Paraganglioma)) OR (Gangliocytic Paragangliomas))
Intervention	Tyrosine Kinase Inhibitors	#3	("Tyrosine Kinase Inhibitors"[Mesh]) OR ((((((Tyrosine Kinase Inhibitors) OR (Inhibitors, Tyrosine Kinase)) OR (Kinase Inhibitors, Tyrosine)) OR (Tyrosine Protein Kinase Inhibitors)) OR (Tyrosine Kinase Inhibitor)) OR (TKI Tyrosine Kinase Inhibitors))
	Sunitinib	#4	("Sunitinib"[Mesh]) OR ((((((((((Sunitinib) OR (5-(5-Fluoro-2-oxo-1,2-dihydroindolylidenemethyl)-2,4-dimethyl-1H-pyrrole-3-carboxylic acid (2-diethylaminoethyl)amide)) OR (SU 11248)) OR (SU11248)) OR (SU-11248)) OR (SU 011248)) OR (SU-011248)) OR (SU011248)) OR (Sunitinib Malate)) OR (Sutent))
	Cabozantinib	#5	("cabozantinib" [Supplementary Concept]) OR ((((((((cabozantinib) OR (XL 184)) OR (XL-184)) OR (XL184 cpd)) OR (BMS 907351)) OR (BMS-907351)) OR (BMS907351)) OR (Cometriq))
	Axitinib	#6	("Axitinib"[Mesh]) OR (((((Axitinib) OR (AG 013736)) OR (AG-013736)) OR (AG013736)) OR (Inlyta))
	Pazopanib	#7	("pazopanib" [Supplementary Concept]) OR ((((((((pazopanib) OR (GW 780604)) OR (GW-780604)) OR (GW780604)) OR (GW 786034B)) OR (GW-786034B)) OR (GW786034B)) OR (Votrient))
	Lenvatinib	#8	("lenvatinib" [Supplementary Concept]) OR ((((((((((((((lenvatinib) OR (4-(3-chloro-4-((cyclopropylaminocarbonyl)amino)phenoxy)-7-methoxy-6-quinolinecarboxamide)) OR (4-(3-chloro-4-(N'-cyclopropylureido)phenoxy)-7-methoxyquinoline-6-carboxamide E 7080)) OR (ER-203492-00)) OR (E7080)) OR (E-7080)) OR (lenvatinib metabolite M2)) OR (4-(3-chloro-4-(((cyclopropylamino)carbonyl)amino)phenoxy)-7-hydroxy-6-quinolinecarboxamide)) OR (lenvatinib mesylate)) OR (lenvatinib mesilate)) OR (lenvatinib methanesulfonate)) OR (N-(4-((6-carbamoyl-7-methoxyquinolin-4-yl)oxy)-2-chlorophenyl)-N'-cyclopropylurea monomethanesulfonate)) OR (E-7080 mesylate)) OR (E7080 mesylate)) OR (Lenvima))
	Anlotinib	#9	("anlotinib" [Supplementary Concept]) OR ((anlotinib) OR (AL3818))

B. Search Strategy in Pubmed Database

Topic	Query	No. of studies
Pheochromocytoma	#1	26897
Paraganglioma	#2	28368
**Patients/disease**	#1 OR #2	36289
Tyrosine Kinase Inhibitors	#3	105924
Sunitinib	#4	8362
Cabozantinib	#5	2274
Axitinib	#6	1967
Pazopanib	#7	2719
Lenvatinib	#8	3847
Anlotinib	#9	1258
**Intervention**	#3 OR #4 OR #5 OR #6 OR #7 OR #8 OR #9	117327
**Final search terms**	(#1 OR #2) AND (#3 OR #4 OR #5 OR #6 OR #7 OR #8 OR #9)	320

C. Search Strategy in Embase Database

Topic	Query	No. of studies
Pheochromocytoma	#1	40038
Paraganglioma	#2	16254
**Patients/disease**	#1 OR #2	49356
Tyrosine Kinase Inhibitors	#3	135,536
Sunitinib	#4	30382
Cabozantinib	#5	8939
Axitinib	#6	8300
Pazopanib	#7	11932
Lenvatinib	#8	8833
Anlotinib	#9	1584
**Intervention**	#3 OR #4 OR #5 OR #6 OR #7 OR #8 OR #9	164939
**Final search terms**	(#1 OR #2) AND (#3 OR #4 OR #5 OR #6 OR #7 OR #8 OR #9)	1021

D. Search strategy in Web of Science Database

Topic	Query	No. of studies
Pheochromocytoma	#1	44832
Paraganglioma	#2	15024
**Patients/disease**	#1 OR #2	54018
Tyrosine Kinase Inhibitors	#3	147,998
Sunitinib	#4	16931
Cabozantinib	#5	3896
Axitinib	#6	3108
Pazopanib	#7	4605
Lenvatinib	#8	4623
Anlotinib	#9	1469
**Intervention**	#3 OR #4 OR #5 OR #6 OR #7 OR #8 OR #9	166377
**Final search terms**	(#1 OR #2) AND (#3 OR #4 OR #5 OR #6 OR #7 OR #8 OR #9)	963

Supplementary Table 2. Eligibility and baseline prognostic characteristics, prior treatment, line of therapy, and outcome analysis in the included studies

Study / agent	Eligibility and baseline progression	Disease burden and metastatic sites	Molecular / functional status	Prior therapy	Line of therapy	Outcome population
Ayala-Ramirez 2012<br>Sunitinib	Progressive disease within <=6 months; retrospective; performance-status threshold NR	11 PHEO/6 sympathetic PGL; LN 12/17, bone 11/17, liver 10/17, lung 6/17; 4 bone-only; one >50% liver involvement	*SDHB* 8; *VHL* 1; functional status NR	Chemotherapy 10/17; MIBG 2/17	Predominantly salvage; exact line NR	14/17 measurable and response-evaluable; ORR 21.4%, DCR 57.1%
O'Kane 2019<br>SNIPP; sunitinib	ECOG 0-2; initially required radiographic/biochemical progression, later amended to include symptomatic disease without radiographic progression	23 metastatic and 2 locally advanced; LN 44%, lung 48%, bone 48%, liver 56%	15 tested; 9 germline variants, including *SDHB* 5; secretory status NR	Prior systemic therapy for advanced disease 3/25; 21/25 had no prior systemic therapy	Mostly first systemic therapy	23/25 measurable and response-evaluable; ORR 13.0%, DCR 82.6%
Nelson 2022<br>Lenvatinib	Retrospective cohort after an aborted phase II study; ECOG <2; prospective subset required measurable RECIST disease	LN and bone 10/11 each; liver/peritoneum 6/11; lung 5/11; 8/11 had measurable disease	*SDHB* 5; secretory status 7/11	All prior CVD; MIBG 5/11; radiotherapy 7/11; prior MKI 2/11	All later line	8/11 measurable and response-evaluable; ORR 62.5%, DCR 100%
Pichun 2015 / registry results<br>Axitinib; NCT01967576	Phase II; ECOG <=2; RECIST-measurable disease; no prior TKI; detailed baseline progression window NR in abstract	Sites and burden NR	Molecular and secretory status NR	Prior therapy allowed after washout; no prior TKI	NR	12/14 measurable and response-evaluable; ORR 41.7%, DCR 83.3%
Baudin 2024<br>FIRSTMAPPP; sunitinib	Randomized placebo-controlled phase II; RECIST progression within 18 months; ECOG 0-2; measurable disease; central blinded review	Bone 27/39; 1, 2, 3, and 4 metastatic sites in 8, 15, 11, and 5 patients	*SDHB* 13/39; baseline hypertension 16/39	Any prior treatment 26/39; MIBG 7, chemotherapy 11, radiotherapy 14, PRRT 2	First line 17/39; later line 22/39	Sunitinib randomized arm n=39; 36/39 response-evaluable; ORR 36.1%, DCR 72.2%
Jimenez 2024<br>Natalie; cabozantinib	Phase II; RECIST progression within 12 months; ECOG 0-2; bone-only disease permitted	10 PHEO/7 PGL; patient-level bone, liver, lung, LN, and peritoneal metastases reported; 16/17 measurable	*SDHB* 5; hormonally active 12/17	11/17 systemic-therapy naive; prior therapies reported at patient level	Mostly first systemic therapy	16/17 measurable and response-evaluable; ORR 25%, DCR 93.8%
Nasca 2024<br>SUTNET; sunitinib	Phase II; radiologically progressive disease or distant metastases; no uniform progression window; ECOG 0-2; measurable RECIST disease	At least 2 metastatic sites in 32/50; bone 29, liver 20, lung 13, LN 13	NGS in 31: pseudohypoxia 17, kinase 7; *SDHB* 9; ECOG 0/1/2: 33/12/5	No prior systemic therapy 28/50; prior therapy 22/50, including MIBG/PRRT 16, chemotherapy 7, SSA 3	First line 28/50; later line 22/50	45/50 measurable and response-evaluable; ORR 15.6%, DCR 68.9%
Park 2024<br>Sunitinib subgroup	Retrospective institutional cohort; sunitinib subgroup n=6; progression criterion and ECOG for subgroup NR	Overall cohort: bone 19/33, LN 14/33, lung 12/33, liver 4/33; recurrent 24, initially metastatic 7, locally advanced 2	23 tested; 9 germline variants, including *SDHB* 5; secretory 23/33	All six sunitinib patients previously received CVD	Second line 5/6; third line 1/6	Sunitinib subgroup n=6; ORR 33.3%, DCR 83.3%
Tian 2024<br>Anlotinib	Retrospective; radiographic and/or biochemical progression, or new symptoms without radiographic progression; measurable disease required for tumor assessment	15 PCC/22 PGL; 36 metastatic, 1 locally advanced; LN 29, bone 27, lung 16, liver 12	Genetics available in 13, including *SDHB* 5; secretory 31/37	Prior surgery 29/37; PRRT 7, chemotherapy 6, MIBG 6, prior sunitinib 3	First line 16/37; second 11/37; third or later 10/37	8/11 measurable and response-evaluable; ORR 42.1%, DCR 94.7%
Cui 2025<br>Anlotinib	Phase II; radiographic progression over prior 3-12 months or new pronounced symptoms; ECOG 0-2	Bone 17/29, LN 12/29, lung 11/29, liver 7/29, soft tissue 6/29	Germline results in 28: *SDHB* 13, *RET* 3, *FH* 1; secretory 24/29	Temozolomide 16/29; local therapy 9/29; MIBG 4/29; treatment-naive 4/29	Mostly previously treated	22/29 measurable and response-evaluable; ORR 31.8%, DCR 95.5%

Study / agent	Eligibility and baseline progression	Disease burden and metastatic sites	Molecular / functional status	Prior therapy	Line of therapy	Outcome population
Fojo 2024<br>Axitinib; NCT03839498	Progressive PPGL required; detailed ECOG, progression window, and measurable-disease criteria NR in one-page abstract	Sites and burden NR	Molecular and secretory status NR	Prior therapy and line NR	NR	17/19 measurable and response-evaluable; ORR 35.3%

Abbreviations: NA, not available; CVD, cyclophosphamide, vincristine, and dacarbazine; DCR, disease control rate; ECOG, Eastern Cooperative Oncology Group performance status; LN, lymph node; MIBG, metaiodobenzylguanidine; MKI, multikinase inhibitor; NGS, next-generation sequencing; NR, not reported; ORR, objective response rate; PCC, pheochromocytoma; PHEO, pheochromocytoma; PGL, paraganglioma; PPGL, pheochromocytoma and paraganglioma; PRRT, peptide receptor radionuclide therapy; RECIST, Response Evaluation Criteria in Solid Tumors; SSA, somatostatin analogue.

Supplementary Table 3. Quality Assessment

A. Joanna Briggs Institute (JBI) Tools for Assessing Case Series.

Author	Year	Study type	Scale	Were there clear criteria for inclusion in the case series?	Was the condition measured in a standard, reliable way for all participants included in the case series?	Were valid methods used for identification of the condition for all participants included in the case series?	Did the case series have consecutive inclusion of participants?	Did the case series have complete inclusion of participants?	Was there clear reporting of the demographics of the participants in the study?	Was there clear reporting of clinical information of the participants?	Were the outcomes or follow-up results of cases clearly reported?	Was there clear reporting of the presenting site(s)/clinic(s) demographic information?	Was statistical analysis appropriate?
Ayala-Ramirez, M	2012	obser	JBI	Yes	Yes	Yes	Yes	Yes	Yes	Yes	Yes	Not applicable	Yes
Nelson, L.H,	2022	obser+inter	JBI	Yes	Yes	Yes	No	No	Yes	Yes	Yes	Not applicable	Yes
Park, Y.G.	2024	obser	JBI	Yes	Yes	Yes	Yes	Yes	Yes	No	Yes	Not applicable	Yes
Tian, R.	2024	obser	JBI	Yes	Yes	Yes	Yes	Yes	Yes	No	Yes	Not applicable	Yes

Abbreviations: obser, observational; inter, interventional.

B. Methodological Index for Non-Randomized Studies (MIONRS).

Author	Year	Study type	Scale	A clearly stated aim	Inclusion of consecutive patients	Prospective collection of data	Endpoints appropriate to the aim of the study	Unbiased assessment of the study endpoint	Follow-up period appropriate to the aim of the study	Loss to follow up less than 5%	Prospective calculation of the study size
Baudin, E.	2024	RCT	MINORs	2	2	2	2	2	2	0	2
Fojo, A.T.	2024	inter	MINORs	2	2	1	1	2	2	0	2
Jimenez, C.	2024	inter	MINORs	2	2	2	1	2	2	0	2
Nasca, V.	2024	inter	MINORs	2	2	2	1	2	2	2	0
O'Kane, G.M	2019	inter	MINORs	2	2	2	1	2	2	0	2
Pichun, M. E. B.	2015	inter	MINORs	2	2	1	1	2	2	0	2
Cui, Y.Y.	2025	inter	MINORs	2	2	1	2	2	2	0	2

Each term is scored as 0 (not reported), 1 (reported but inadequate), 2 (reported and adequate). Abbreviations: inter, interventional; RCT, randomized controlled trial.

Supplementary Table 4. Tumor Response and Survival Data

First author	Year	Tumor response							Survival data				
		Patients available	TKIs	CR, No. (%)	PR, No. (%)	SD, No. (%)	PD, No. (%)	DCR, %	Patients available	mOS, m (95% CI)	OS at 12 months (95% CI)	Median PFS, m (95% CI)	PFS at 12 months (95% CI)
Ayala-Ramirez, M.	2012	14	Sunitinib	0	3(21.4)	5(35.7)	6(42.9)	57.1	17	26.7	NA	4.1 (1.4-11.0)	NA
Baudin, E.	2024	36	Sunitinib	0	13(36.1)	13(36.1)	10(27.8)	72.2	39	26·1 (18·7-36·7)	78·3% (62·6–88·6)	8.9 (5.5-12.7)	35·9% (22·7–51·6)
Fojo, A.T.	2024	17	Axitinib	NA	6(35.3)	NA	NA	NA	17	29	NA	7.9	NA
Jimenez, C.	2024	16	Cabozantinib	0	4(25.0)	11(68.8)	1(6.3)	93.8	17	24·9 (23·6–not reached)	NA	16.6 (8.1–34.9)	58.8%（39.5–87.6）
Nasca, V.	2024	45	Sunitinib	0	7(15.6)	24 (53.3)	14 (31.1)	68.9	50	49.4 (21.2-NA)	75.4% (64.1-88.5)	14.1 (8.9–25.7)	53.4% (41.1–69.3)
Nelson, L.H,	2022	8	Lenvatinib	0	5(62.5)	3(37.5)	0	100.0	11	n.rea	80.8% (42.3-.94.9)	14.7 (1.7-NE)	61.4% (26.6-83.5)
O'Kane, G.M.	2019	23	Sunitinib	0	3(13.0)	16(69.6)	4(17.4)	82.6	25	n.rea	NA	13.4 (5.3-24.6)	NA
Park, Y.G.	2024	6	Sunitinib	0	2(33.3)	3(50)	1(16.7)	83.3	6	14.6 (3.6–NA)	NA	14.6 (2.7–NA)	NA
Pichun, M. E. B.	2015	12	Axitinib	0	5(41.7)	5(41.7)	2(16.7)	83.3	12	NA	NA	7.7 (3.3-16.8)	NA
Tian, R.	2024	19	Anlotinib	0	8(42.1)	10(52.6)	1(5.3)	94.7	NA	NA	NA	NA	NA
Cui, Y.Y.	2025	22	Anlotinib	0	7(31.8)	14(63.6)	1(4.5)	95.5	22	NA	NA	22.6(19.4-25.8)	NA

Abbreviations: NA, not available; n.rea, not reached; mOS, median overall survival; m, months; CR, complete response; DCR, disease control rate; ORR, objective response rate; OS, overall survival; PD, progressive disease; PFS, progression-free survival; PR, partial response; SD, stable disease.

Supplementary Table 5. Safety of TKI Treatment

First author	Year	Patients evaluable, No.	Most commonly reported AEs (No. or %)	Most commonly reported Grade 1-2 (No.)	Most commonly reported Grade 3-4 (No.)	Most commonly reported Grade 5 (No.)	Patients discontinued or decreased drug due to AEs (No.)
Ayala-Ramirez, M.	2012	17	Hypertension (NA)<br>Diarrhea (NA)<br>PPES (NA)<br>Sore mouth (NA)<br>Fatigue (NA)	NA	Grade 3: intolerable pain of bone metastases (1), frequent syncope (1), intolerable fatigue (1), and hand-foot syndrome (1);<br>Grade 4: hypertensive crisis with pulmonary edema (1).	NA	Discontinued: 4<br>Decreased: 2
Baudin, E.	2024	39	Diarrhea (26)<br>Asthenia (28)<br>Weight loss (18)<br>Vomiting (16)	Diarrhea (25)<br>Asthenia (21)<br>Weight loss (18)<br>Vomiting (15)	Asthenia (7)<br>Hypertension (5)	Amyotrophic lateral sclerosis (1)<br>Rectal bleeding (1)<br>Shortness of breath (1)	Discontinued: 5<br>Decreased: 17
Fojo, A.T.	2024	17	Grade 1-2: fatigue, diarrhea, mucositis, and PPE (42%-63%);<br>G1/2/3: hypertension (79%)	NA	NA	NA	Discontinued: 0
Jimenez, C.	2024	17	Fatigue (16)<br>Hypertension (16)<br>Increased ALT or AST (15)	Fatigue (16)<br>Increased ALT or AST (15)<br>Hypertension (15)	Grade 3: increased amylase or lipase (2), hypomagnesaemia (1), hand-and-foot syndrome (1), hypertension (1), rectal fistula (1), QT interval prolongation (1).	NA	Discontinued: 0<br>Decreased: 15
Nasca, V.	2024	50	Fatigue (33)<br>Hypertension (25)<br>Mucositis (24)	Fatigue (30)<br>Hypertension (19)<br>Mucositis (17)	Mucositis (7)<br>Hypertension (6)<br>Myelosuppression (6)	Brain hemorrhage (1)	Discontinued: 5<br>Decreased: 34
Nelson, L.H,	2022	11	Hypertension (9)	NA	NA	NA	Discontinued: 2<br>Decreased: 10
O'Kane, G.M.	2019	25	Fatigue (17)<br>Nausea/vomiting (16)<br>PPES (16)<br>Diarrhea (11)	Fatigue (13)<br>Nausea and/or vomiting (14)<br>PPES (14)	Grade 3 or 4: fatigue (4), thrombocytopenia (4), nausea/vomiting (2), PPES (2), hypertension (2), anaemia (2), hyponatremia (2), diarrhea (1), mucositis (1), AST/ALT increased (1), neutropenia (1), cardiac ischaemia (1), cardiomyopathy (1).	NA	Discontinued: 5<br>Decreased: 14
Pichun, M. E. B.	2015	14	Hypertension (14)<br>Fatigue (13)<br>Lymphocyte counts decreased (12)<br>Anaemia (10)	NA	Serious AE (2)	NA	Discontinued: 1
Tian, R.	2024	37	Malaise (25)<br>Hypertension (25)<br>PPES (24)<br>Oral mucositis (20)	Malaise (24)<br>PPES (21)<br>Oropharyngeal pain (19)	Hypertension (23)<br>PPES (3)<br>Diarrhea (2)<br>Malaise (1)<br>Oropharyngeal pain (1)<br>Bleeding (1)<br>Pain (1)	NA	Discontinued: 1<br>Decreased: 5
Cui, Y.Y.	2025	29	Hypertension (24)<br>Subclinical primary hypothyroidism (11)<br>Hyperlipidemia (10)	Hypertension (23)<br>Subclinical primary hypothyroidism (11)<br>Hyperlipidemia (10)	Hypertension (1)	NA	Discontinued: 7

Abbreviations: NA, not available; AE, adverse event; PPES, palmar–planter erythrodysesthesia syndrome.

Supplementary Figure 1. Forest Plot of Tumor Response for Patients with *SDHB* and with Non-*SDHB* PPGLs

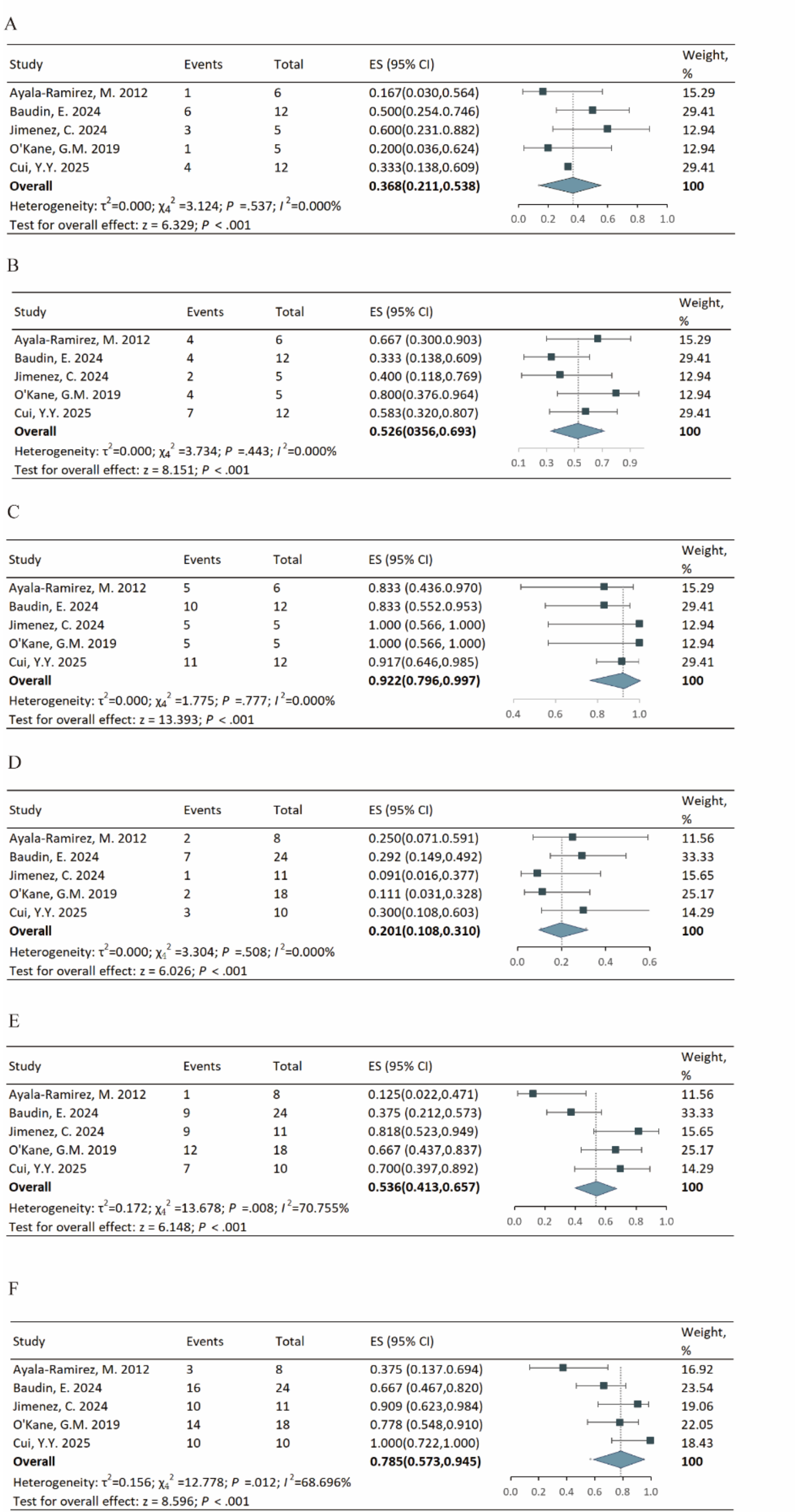

A

Study	Events	Total	ES (95% CI)	Weight, %
Ayala-Ramirez, M. 2012	1	6	0.167(0.030,0.564)	15.29
Baudin, E. 2024	6	12	0.500(0.254.0.746)	29.41
Jimenez, C. 2024	3	5	0.600(0.231.0.882)	12.94
O'Kane, G.M. 2019	1	5	0.200(0.036,0.624)	12.94
Cui, Y.Y. 2025	4	12	0.333(0.138,0.609)	29.41
**Overall**			**0.368(0.211,0.538)**	**100**

Heterogeneity: τ^2=0.000; χ_4^2 =3.124; *P* =.537; I^2=0.000%

Test for overall effect: z = 6.329; *P* < .001

B

Study	Events	Total	ES (95% CI)	Weight, %
Ayala-Ramirez, M. 2012	4	6	0.667 (0.300.0.903)	15.29
Baudin, E. 2024	4	12	0.333 (0.138,0.609)	29.41
Jimenez, C. 2024	2	5	0.400 (0.118,0.769)	12.94
O'Kane, G.M. 2019	4	5	0.800(0.376.0.964)	12.94
Cui, Y.Y. 2025	7	12	0.583(0.320,0.807)	29.41
**Overall**			**0.526(0356,0.693)**	**100**

Heterogeneity: τ^2=0.000; χ_4^2 =3.734; *P* =.443; I^2=0.000%

Test for overall effect: z = 8.151; *P* < .001

C

Study	Events	Total	ES (95% CI)	Weight, %
Ayala-Ramirez, M. 2012	5	6	0.833 (0.436.0.970)	15.29
Baudin, E. 2024	10	12	0.833 (0.552.0.953)	29.41
Jimenez, C. 2024	5	5	1.000 (0.566, 1.000)	12.94
O'Kane, G.M. 2019	5	5	1.000 (0.566, 1.000)	12.94
Cui, Y.Y. 2025	11	12	0.917(0.646,0.985)	29.41
**Overall**			**0.922(0.796,0.997)**	**100**

Heterogeneity: τ^2=0.000; χ_4^2 =1.775; *P* =.777; I^2=0.000%

Test for overall effect: z = 13.393; *P* < .001

D

Study	Events	Total	ES (95% CI)	Weight, %
Ayala-Ramirez, M. 2012	2	8	0.250(0.071.0.591)	11.56
Baudin, E. 2024	7	24	0.292 (0.149,0.492)	33.33
Jimenez, C. 2024	1	11	0.091(0.016,0.377)	15.65
O'Kane, G.M. 2019	2	18	0.111 (0.031,0.328)	25.17
Cui, Y.Y. 2025	3	10	0.300(0.108,0.603)	14.29
**Overall**			**0.201(0.108,0.310)**	**100**

Heterogeneity: τ^2=0.000; χ_4^2 =3.304; *P* =.508; I^2=0.000%

Test for overall effect: z = 6.026; *P* < .001

E

Study	Events	Total	ES (95% CI)	Weight, %
Ayala-Ramirez, M. 2012	1	8	0.125(0.022,0.471)	11.56
Baudin, E. 2024	9	24	0.375 (0.212,0.573)	33.33
Jimenez, C. 2024	9	11	0.818(0.523,0.949)	15.65
O'Kane, G.M. 2019	12	18	0.667 (0.437,0.837)	25.17
Cui, Y.Y. 2025	7	10	0.700(0.397,0.892)	14.29
**Overall**			**0.536(0.413,0.657)**	**100**

Heterogeneity: τ^2=0.172; χ_4^2 =13.678; *P* =.008; I^2=70.755%

Test for overall effect: z = 6.148; *P* < .001

F

Study	Events	Total	ES (95% CI)	Weight, %
Ayala-Ramirez, M. 2012	3	8	0.375 (0.137.0.694)	16.92
Baudin, E. 2024	16	24	0.667 (0.467,0.820)	23.54
Jimenez, C. 2024	10	11	0.909 (0.623,0.984)	19.06
O'Kane, G.M. 2019	14	18	0.778 (0.548,0.910)	22.05
Cui, Y.Y. 2025	10	10	1.000(0.722,1.000)	18.43
**Overall**			**0.785(0.573,0.945)**	**100**

Heterogeneity: τ^2=0.156; χ_4^2 =12.778; *P* =.012; I^2=68.696%

Test for overall effect: z = 8.596; *P* < .001

(A) *SDHB*-PR; (B) *SDHB*-SD; (C) *SDHB*-DCR; (D) Non-*SDHB*-PR; (E) Non-*SDHB*-SD; (F) Non-*SDHB*-DCR

Supplementary Figure 2. Funnel plot and Egger regression plot of PR/ORR

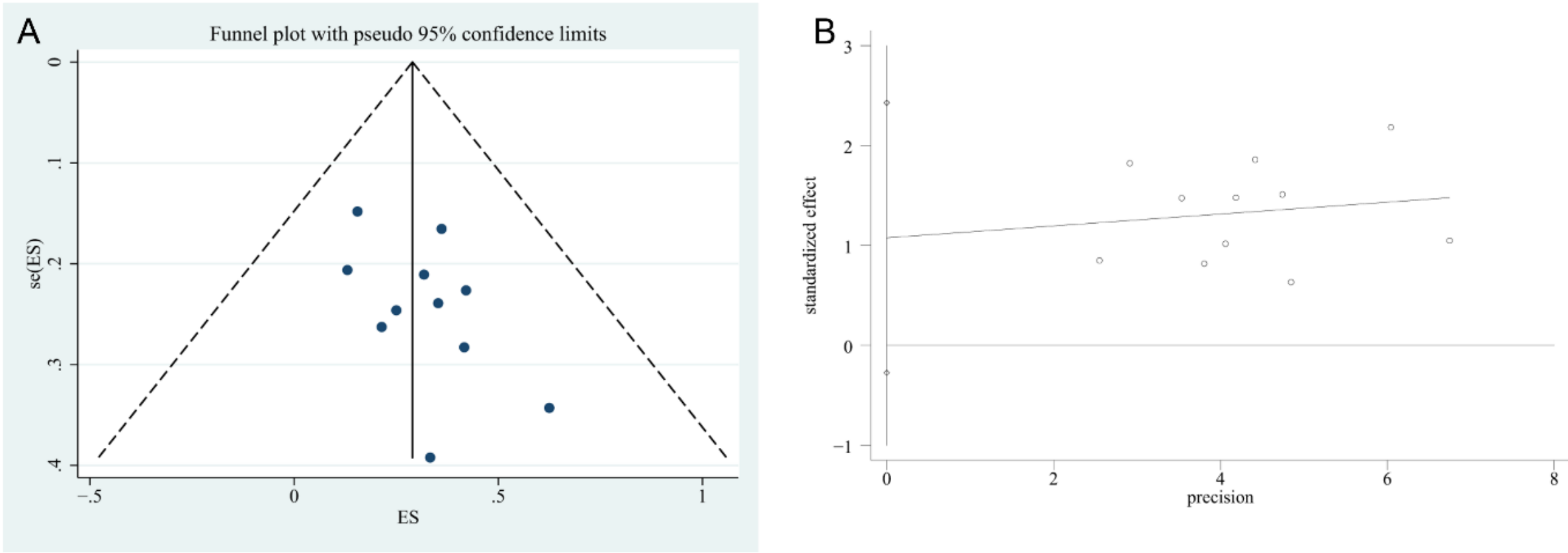

(A) The funnel plot of partial response (PR)/objective response rate (ORR) in included studies. (B) The Egger's test assessing funnel-plot asymmetry.

Supplementary Figure 3: Funnel plot and Egger regression plot of SD

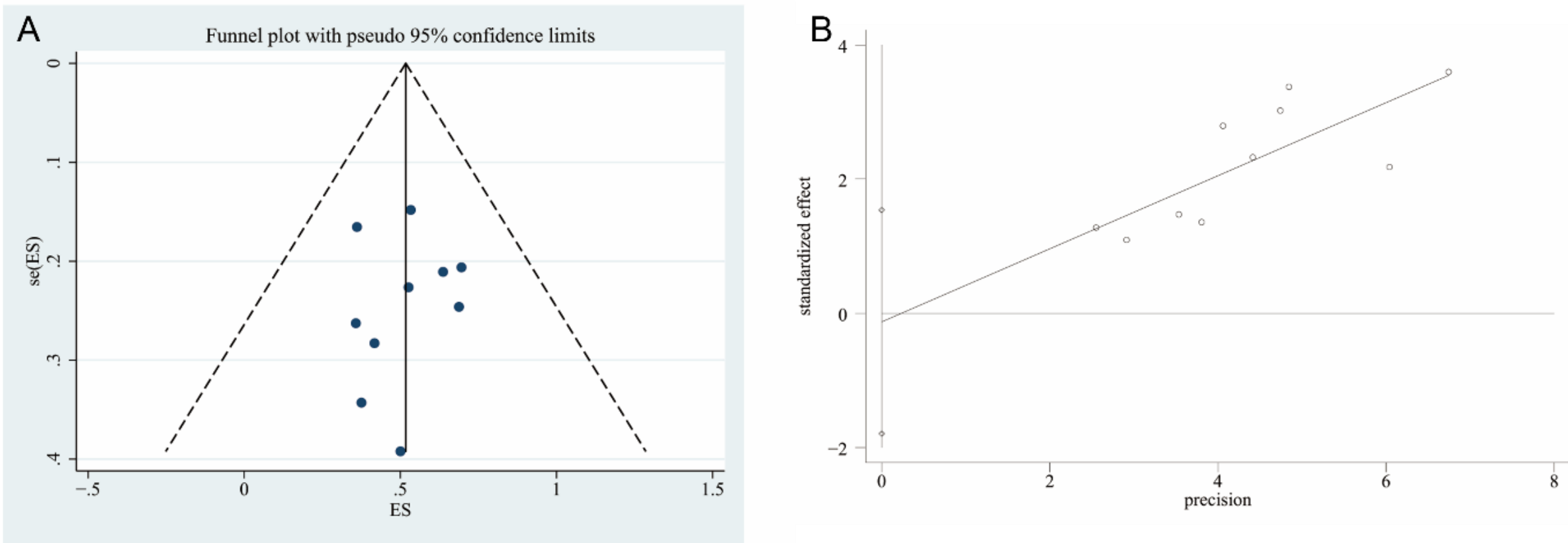

(A) The funnel plot of stable disease (SD) in included studies. (B) The Egger's test assessing funnel-plot asymmetry.

Supplementary Figure 4: Funnel plot and Egger regression plot of DCR

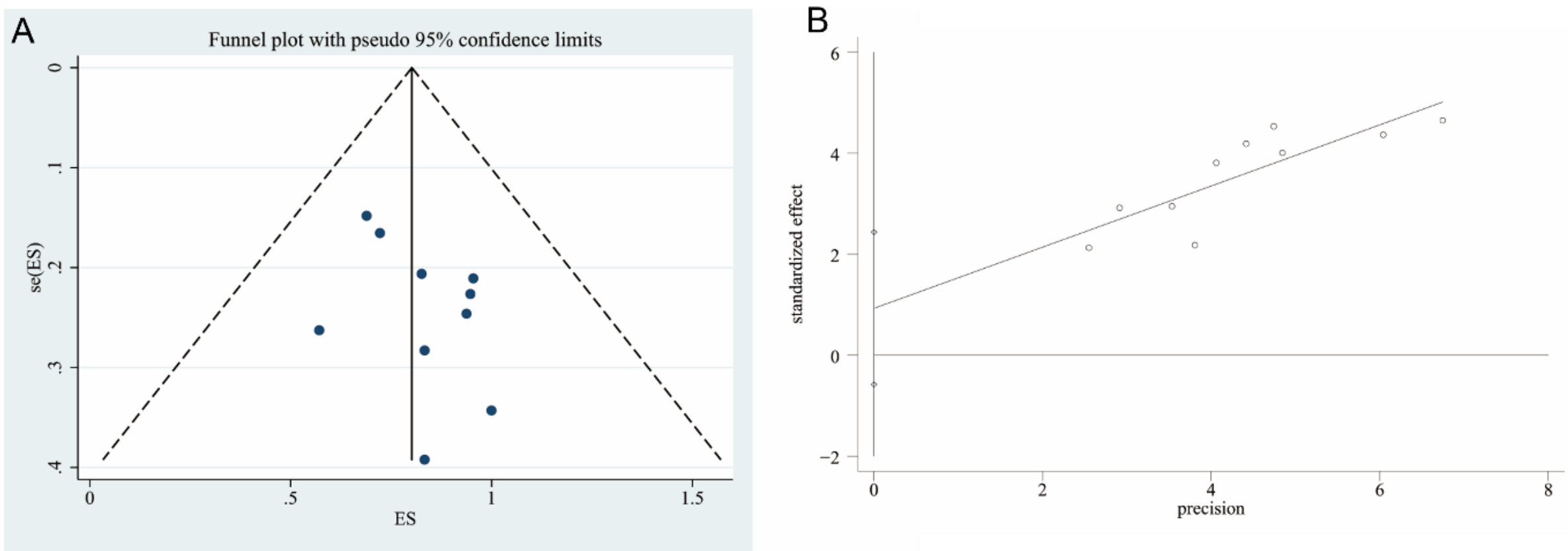

(A) The funnel plot of disease control rate (DCR) in included studies. (B) The Egger's test assessing funnel-plot asymmetry.

Supplementary Figure 5. Funnel plot and Egger regression plot of AEs

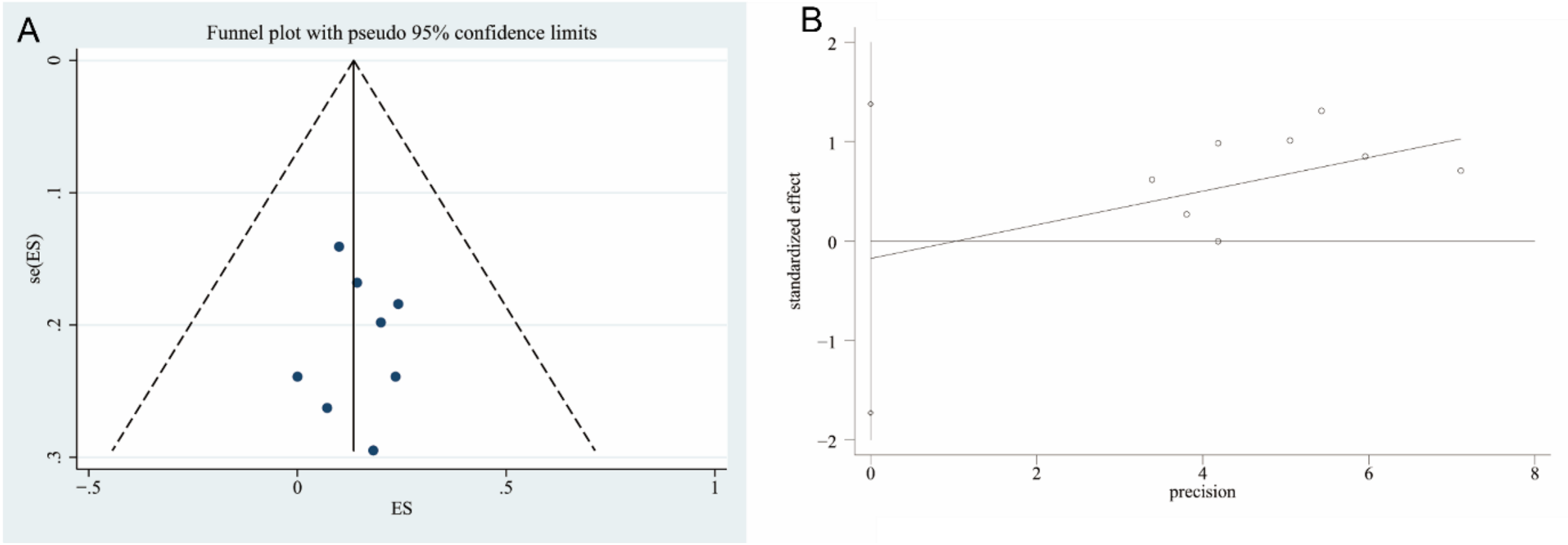

(A) The funnel plot of adverse events (AEs) in included studies. (B) The Egger's test assessing funnel-plot asymmetry.

Supplementary Figure 6. Sensitivity Analysis

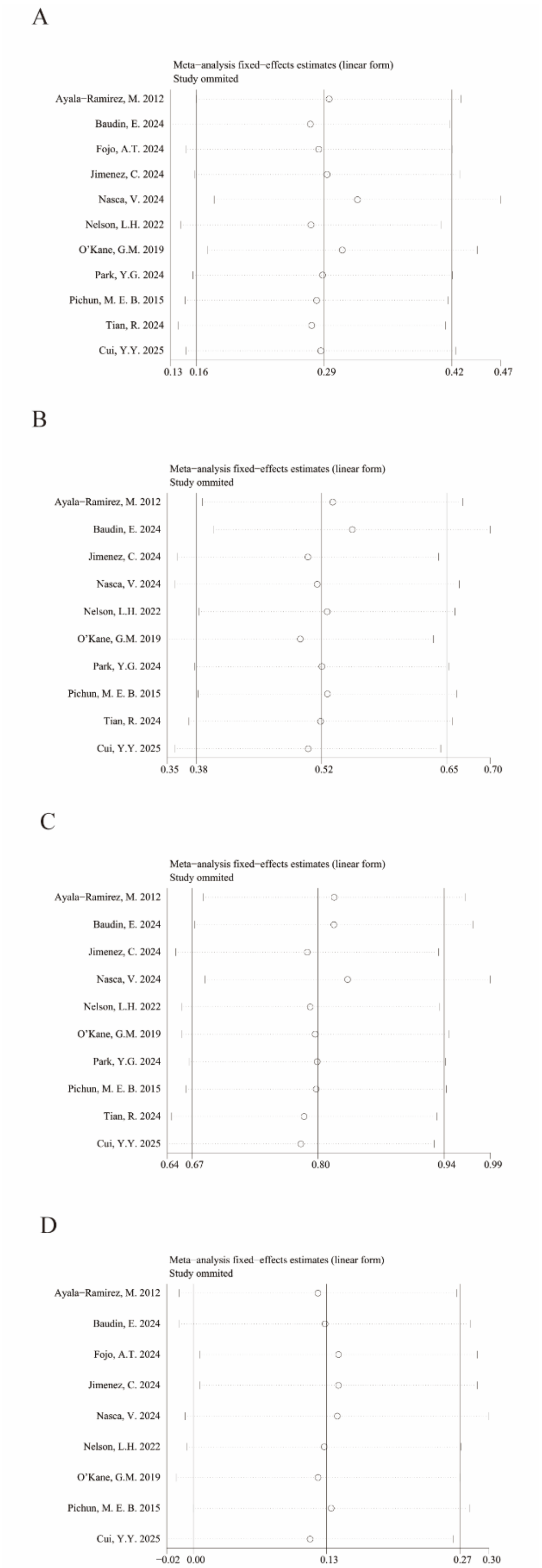

The forest plot of the sensitivity analysis of the pooled proportion of partial response (A), stable disease (B), disease control rate (C), and adverse events (D) through recalculating the pooled effects after omitting each single study.
